# Randomized, Open-Label Phase 2 Study of Apalutamide plus Androgen Deprivation Therapy versus Apalutamide Monotherapy versus Androgen Deprivation Monotherapy in Patients with Biochemically Recurrent Prostate Cancer

**DOI:** 10.1155/2022/5454727

**Published:** 2022-09-28

**Authors:** Rahul Aggarwal, Joshi J. Alumkal, Russell Z. Szmulewitz, Celestia S. Higano, Alan H. Bryce, Angela Lopez-Gitlitz, Sharon A. McCarthy, Branko Miladinovic, Kelly McQuarrie, Shibu Thomas, Ke Zhang, Eric J. Small

**Affiliations:** ^1^Helen Diller Family Comprehensive Cancer Center, University of California San Francisco, San Francisco, CA, USA; ^2^Knight Cancer Institute, Oregon Health and Science University, Portland, OR, USA; ^3^Rogel Cancer Center, University of Michigan, Ann Arbor, MI, USA; ^4^University of Chicago, Chicago, IL, USA; ^5^University of Washington, Fred Hutchinson Cancer Research Center, Seattle, WA, USA; ^6^Mayo Clinic Arizona, Scottsdale, AZ, USA; ^7^Janssen Research & Development, Los Angeles, CA, USA; ^8^Janssen Research & Development, Raritan, NJ, USA; ^9^Janssen Research & Development, San Diego, CA, USA; ^10^Merck, Rahway, NJ, USA; ^11^Janssen Research & Development, Spring House, PA, USA

## Abstract

**Purpose:**

This randomized phase 2 study sought to assess the treatment effect of a finite duration of apalutamide with and without androgen deprivation therapy (ADT) in biochemically recurrent prostate cancer (BCR PC)*. Materials and Methods*. Patients with BCR PC after primary definitive therapy and prostate-specific antigen (PSA) doubling time ≤12 months were randomized to open-label apalutamide (240 mg/d) alone, apalutamide plus ADT, or ADT alone (1 : 1:1 ratio) for 12 months followed by a 12-month observation period (NCT01790126). Mean changes from baseline in Functional Assessment of Cancer Therapy-Prostate (FACT-P) at 12 months (primary endpoint) and other prespecified assessments of health-related quality of life (HRQoL), PSA nadir, time to PSA progression, time to testosterone recovery, recovered testosterone >150 ng/dL without PSA progression at 24 months, and molecular markers were evaluated.

**Results:**

In 90 enrolled patients (apalutamide plus ADT (*n* = 31), apalutamide (*n* = 29), ADT (*n* = 30)), FACT-P at 12 months was not significantly different between apalutamide, ADT and apalutamide, and ADT groups. Addition of apalutamide to ADT prolonged time to PSA progression but this change did not reach statistical significance (hazard ratio (HR): 0.56, 95% confidence interval (CI): 0.23–1.36, *P*=0.196); time to testosterone recovery was similar in the ADT-containing groups. In apalutamide plus ADT, apalutamide, and ADT groups, 37.9%, 37.0%, and 19.2% of patients, respectively, had testosterone >150 ng/dL at 24 months without confirmed PSA progression. Of the few biomarkers expressed in blood, *EPHA3* was significantly associated with shorter time to PSA progression (*P*=0.02) in the overall population.

**Conclusions:**

HRQoL was similar in patients treated with apalutamide alone, ADT alone, or their combination, although apalutamide plus ADT did not demonstrate statistically significant noninferiority in change from baseline in overall HRQoL. The aggregated efficacy and safety outcomes support further evaluation of apalutamide plus ADT in BCR PC.

## 1. Introduction

Androgen deprivation therapy (ADT) has long been the standard-of-care treatment for patients with advanced prostate cancer (PC) [1] and is often utilized in patients with biochemically recurrent (BCR) PC with rising prostate-specific antigen (PSA) after completion of definitive/or and salvage local therapy. Despite possible associations with adverse effects and reduced health-related quality of life (HRQoL) in otherwise asymptomatic patients [2, 3], ADT is used widely in BCR PC [4, 5]. ADT-sparing approaches are being investigated [6]. Intermittent ADT has been recommended in patients with high-risk BCR PC [7] because of its similar efficacy with continuous ADT [8, 9] and possible improvements in HRQoL [10] in men with rising PSA after definitive radiotherapy and no evidence of metastatic disease [11]. Prior studies of intermittent ADT have investigated duration of ADT induction ranging from 6 to 12 months [12, 13].

Apalutamide is an oral nonsteroidal androgen receptor (AR) inhibitor approved for nonmetastatic castration-resistant PC and metastatic castration-sensitive PC (mCSPC) in combination with ADT [14, 15]. Apalutamide has been studied in early disease [16–19], but its effect on HRQoL in BCR nonmetastatic CSPC remains unknown. No prior studies in BCR PC have reported the treatment effect of non-castrating next-generation AR inhibitors with or without ADT in a randomized fashion after a finite therapy period. We hypothesized that a 12-month finite treatment duration (1) with apalutamide monotherapy will preserve HRQoL to a greater extent than ADT and (2) with apalutamide combined with ADT will not worsen HRQoL compared with ADT monotherapy. We assessed the effect of apalutamide alone and in combination with ADT on the total score of Functional Assessment of Cancer Therapy-Prostate (FACT-P; an established HRQoL instrument used in clinical studies of patients with advanced PC [20]) at 12 months. We also assessed the results of these interventions with other HRQoL instruments, and with regard to PSA progression, and testosterone recovery. Exploratory analysis of potential circulating biomarkers was undertaken to evaluate possible molecular mechanisms of recurrence and prognostic biomarkers in BCR PC that are currently unknown.

## 2. Materials and Methods

### 2.1. Study Design and Patients

**Figure 1:**
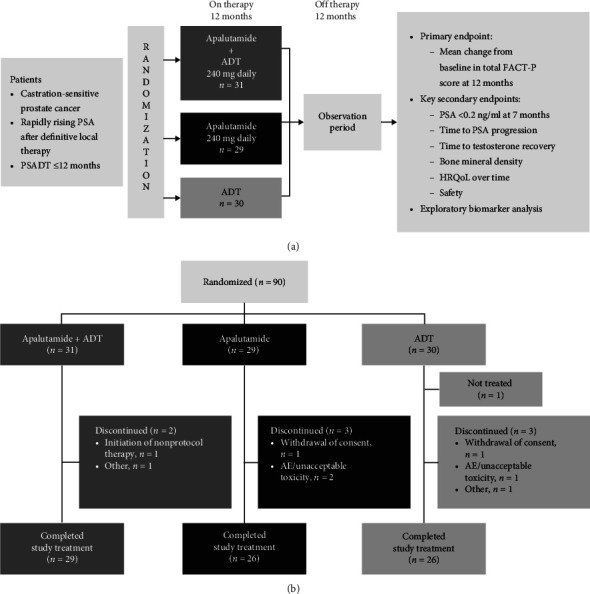
Study design (a) and CONSORT diagram (b) for study ARN-509-002. Stratification factors for randomization: PSADT (<6 versus 6–12 months) and age (≤70 versus >70 years).

This was a phase 2, randomized, open-label, three-group, multicenter study assessing apalutamide (started at 240 mg/d), ADT (luteinizing hormone–releasing hormone agonist), or both (1:1:1 ratio) for 12 months, followed by a 12-month observation period off therapy in patients stratified by PSA doubling time ((PSADT) <6 versus 6–12 months) and age (≤70 versus >70 years) (Figure 1(a)).

The study was conducted at five US sites between February 11, 2013, and March 28, 2019, after gaining approval from institutional review boards. All patients provided written informed consent. Inclusion and exclusion criteria are listed in Table 1.

### 2.2. Outcomes

The primary endpoint was mean change from baseline to 12 months in FACT-P total score (Table 2). The two main objectives of the study were to compare HRQoL as measured by the total FACT-P score at 12 months to assess: (1) noninferiority of apalutamide plus ADT versus ADT monotherapy and (2) superiority of apalutamide monotherapy versus ADT monotherapy. The noninferiority margin was −7, which is within the range of clinically meaningful change of 6 to 10 points reported for advanced PC [23] and 5 to 9 points for other cancer types [24, 25].

Prespecified secondary and exploratory endpoints are summarized in Table 2. Testosterone recovery using threshold testosterone levels >250 ng/dL was assessed.

Treatment-emergent AEs (TEAEs) were reported with onset during the on-therapy period and up to 30 days after the last dose of study medication for each treatment group.

Two exploratory biomarker analyses were performed: one using three molecular classifiers (androgen receptor, DECIPHER,® and PAM50) and archival tumor tissue samples and a second using a custom 36-gene biomarker panel and blood samples collected at baseline and end of the study treatment (12 months of treatment or progression). Details on study design, outcomes, procedures, and statistical analysis as well as additional details on biomarker analysis are in the Supplementary Methods (see S1).

## 3. Results

### 3.1. Patients

Ninety patients were enrolled and randomized (intent-to-treat population): 31 to apalutamide plus ADT, 29 to apalutamide alone, and 30 to ADT alone (Figure 1(a)). One patient randomized to ADT was not treated because of consent withdrawal. Protocol-defined 12-month treatment was completed by 93.5%, 89.7%, and 86.7% of patients in the apalutamide plus ADT, apalutamide, and ADT groups, respectively (Figure 1(b)). Baseline characteristics were similar across treatment groups, with approximately two thirds of patients having <6 months' PSADT at study entry and the majority (>58%) both prior radical prostatectomy and salvage radiation therapy (Table 3).

### 3.2. Health-Related Quality of Life

**Figure 2:**
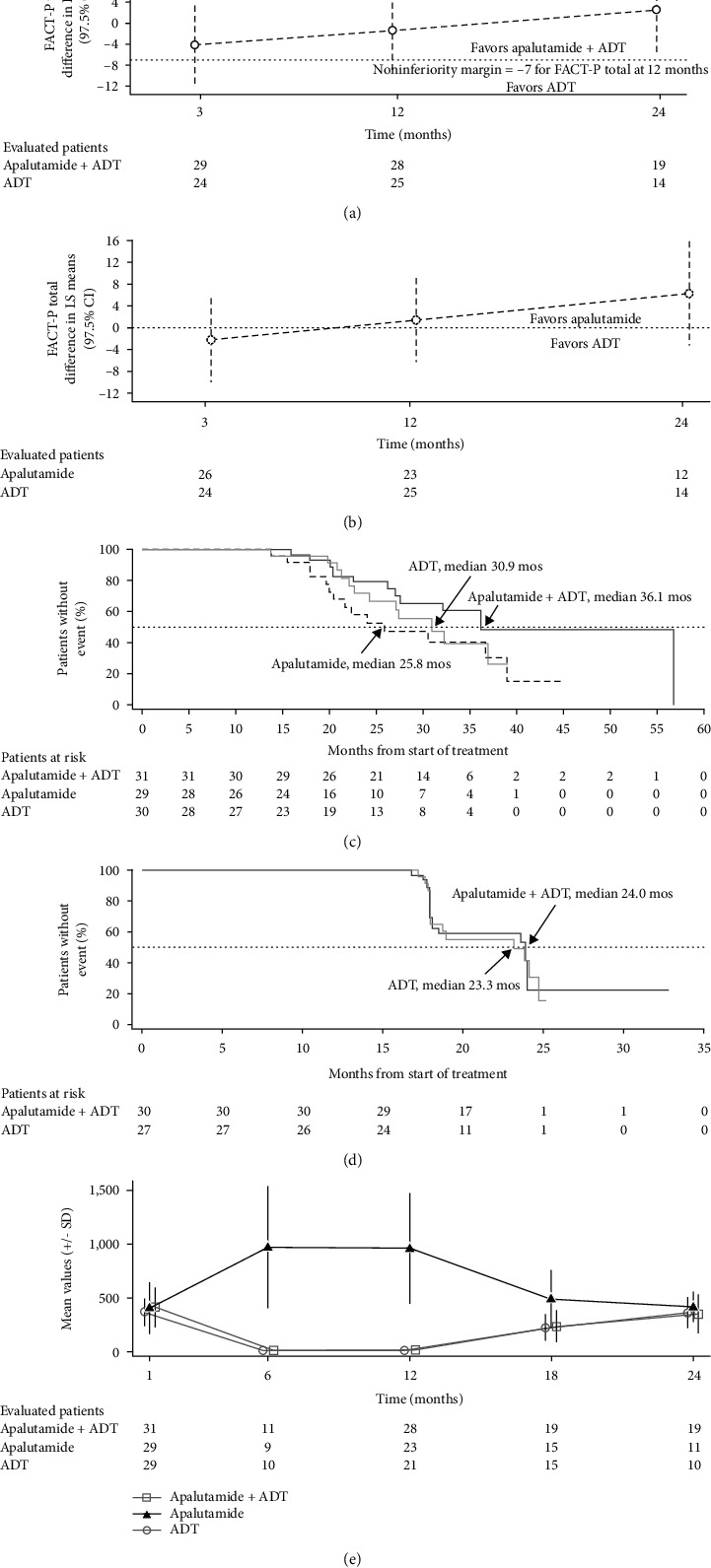
(a) Difference between LS mean change from baseline in FACT-P total scores in apalutamide plus ADT and ADT monotherapy groups. LS mean difference <0 favors ADT. (b) Difference between LS mean change from baseline in FACT-P total scores between apalutamide monotherapy and ADT monotherapy groups. LS mean difference <0 favors ADT. (c) Median time to PSA progression. (d) Median time to serum testosterone (*T*) recovery (*T* > 150 ng/dL). (e) Mean T levels over time.

No significant difference in least squares (LS) mean change from baseline in total FACT-P between apalutamide plus ADT and ADT groups was seen at 12 months (Figure 2(a)). The difference in LS mean was −1.38 (two-sided 97.5% confidence interval (CI): −8.72 to 5.97). As shown in Figure 2(a), because the lower boundary of the CI crossed the prespecified margin of −7 at the 12-month time point, noninferiority of apalutamide plus ADT versus ADT monotherapy with respect to HRQoL was not established.

No significant difference in LS mean change from baseline in total FACT-P between apalutamide monotherapy and ADT monotherapy was seen at 12 months (Figure 2(b)); the LS mean difference of 1.45 (two-sided 97.5% CI: −6.23 to 9.12, *P*=0.669) was also not clinically meaningful, suggesting that apalutamide monotherapy was not superior to ADT monotherapy. The difference in LS mean change from baseline in total FACT-P between apalutamide monotherapy and ADT monotherapy appeared to favor apalutamide over ADT from 3 to 24 months without reaching significance.

The absolute values of FACT-P over time were similar across the treatment groups (Supplementary Figure S1).

No clinically relevant differences in estimated LS mean change from baseline in European Organization for Research and Treatment of Cancer (EORTC) Quality of Life Questionnaire-C30 (QLQ-C30) combined with EORTC QLQ Prostate Cancer Module (PR25) [27] and Sexual Health Inventory for Men (SHIM) [28] over time were observed (Supplementary Table S1).

### 3.3. PSA Nadir and PSA Progression

The median follow-up time for PSA progression was 33.1, 31.4, and 29.8 months for apalutamide plus ADT, apalutamide, and ADT, respectively. Adding apalutamide to ADT resulted in: (1) a higher proportion of patients achieving PSA <0.2 ng/mL at 7 months (96.6%; *n* = 28/29) over apalutamide alone (88.9%; *n* = 24/27) or ADT alone (88.5%; *n* = 23/26) and (2) a lower proportion of patients with PSA progression following discontinuation of combination therapy (38.7%; *n* = 12/31) over apalutamide (48.3%; *n* = 14/29) and ADT (40.0%; *n* = 12/30); however, these differences were not statistically significant. A longer median time to PSA progression for apalutamide plus ADT (36.1 months) over apalutamide (25.8 months) and ADT (30.9 months) was observed (Figure 2(c)); however, the difference between apalutamide plus ADT versus ADT alone did not reach statistical significance (hazard ratio (HR): 0.56, 95% CI: 0.23–1.36, *P*=0.196).

Median time to PSA progression was similar with apalutamide and ADT monotherapies (HR: 1.09, 95% CI: 0.49–2.43, nominal *P*=0.824). Adding apalutamide to ADT resulted in a longer time to PSA progression versus apalutamide monotherapy (HR: 0.40, 95% CI: 0.17–0.98, *P*=0.038) in a post hoc analysis.

### 3.4. Time to Testosterone Recovery

Time to testosterone recovery (testosterone level >150 ng/dL) was similar between apalutamide plus ADT and ADT alone groups (Figure 2(d), Table 4). Testosterone levels in apalutamide-treated patients were supraphysiological during 12 months of treatment, consistent with the mechanism of action of apalutamide, and returned to baseline by 24 months off treatment (Figure 2(e)). At 24 months, 16/19 (84.2%), 10/11 (90.9%), and 9/10 (90.0%) patients had serum testosterone levels >150 ng/dL in the apalutamide plus ADT, apalutamide, and ADT groups, respectively. The addition of apalutamide to ADT resulted in a higher proportion of patients with serum testosterone >150 ng/dL and without PSA progression at 24 months (37.9%) than with ADT alone (19.2%), although the difference was not statistically significant (*P* = 0.147) (Table 4). Apalutamide monotherapy also resulted in a higher proportion of patients with serum testosterone >150 ng/dL without PSA progression at 24 months (37.0%) than that of ADT monotherapy without reaching statistical significance (*P* = 0.169) (Table 4).

Time to testosterone recovery defined as testosterone levels >250 ng/dL (post hoc analysis) was also similar between the apalutamide plus ADT and ADT alone groups (24.0 and 24.1 months, respectively). The proportion of patients with testosterone levels >250 ng/dL and without PSA progression at 24 months was higher in the apalutamide plus ADT and apalutamide monotherapy groups (31.0% and 37.0%, respectively) than that in the ADT monotherapy group (11.5%), without reaching statistical significance (*P*=1.0 and 0.142 for apalutamide plus ADT versus ADT and apalutamide versus ADT).

### 3.5. Bone Mineral Density

No clinically relevant changes in bone mineral density of the femoral neck or lumbar spine assessments at 12 months were observed in any treatment group (Supplementary Table S2).

### 3.6. Safety and Adverse Events

All patients in the apalutamide and apalutamide plus ADT groups and 96.6% in the ADT group reported a TEAE (Table 5). Grade ≥3 TEAEs were more common in the apalutamide plus ADT group (29.0%) than in the apalutamide (17.2%) and ADT groups (13.8%), but there was no discontinuation of the study drug in any treatment group. Individual grade ≥3 TEAEs occurred in one or fewer patients (3.2%–3.4%) in each treatment group, except hypertension, which was reported in four (12.9%) patients treated with apalutamide plus ADT. Treatment-related grade 3 TEAEs occurred in two (6.5%) patients (one with fatigue, one with both hypertension and hypertriglyceridemia) in the apalutamide plus ADT group and in two (6.9%) (both with rash) in the apalutamide group. No grade 4 TEAEs were reported. One death occurred within 30 days of the last dose of the study drug and one death occurred off treatment, both in the ADT group. Notably, gynecomastia and nipple pain were more frequent and falls were less frequent in the apalutamide monotherapy group than in the other groups (Table 5).

Four patients (three in the ADT group and one in the apalutamide monotherapy group) developed radiographic progression based on investigator's assessment of new metastases on conventional imaging.

Dose reductions due to TEAEs and dose interruptions were infrequent (see S2. Supplementary Results).

### 3.7. Biomarker Analysis of Patients from All Treatment Groups

In total, 40 baseline and 54 end-of-study-treatment (EOST; 12 months of treatment or progression) blood samples from patients across all treatment groups were analyzed for biomarkers known for associations with poor prognosis and aggressive phenotype. One patient (2.5%) had AR splice variant ARv7 detected at baseline and EOST. Two patients (3.7%) had ARv7 expressed at EOST. Thirteen of 36 markers were expressed in ≥10% of patients at baseline (Supplementary Table S3). Overall, biomarker prevalence was similar between baseline and EOST, except for *MYBPC1*, *NPY*, and *PGR* transcripts, which were detected in five (12.5%), seven (17.5%), and one (2.5%) patients at baseline and in 12 (22.2%), 14 (25.9%), and six (11.1%) at EOST, respectively. *EPHA3* expression, detected in 12 (30%) patients at baseline and 19 (35.2%) patients at EOST, was the only biomarker whose detection at baseline was significantly associated with shorter time to PSA progression from pooled patients in all three treatment groups (*P*=0.02).

In 26 archival samples, AR activity (AR-A) low, basal, and genomic classifier (GC) high subtypes occurred in 34.6%, 57.7%, and 57.7% of patients, respectively. Across assessed molecular classifiers, the median time to PSA progression was numerically longer only in the AR-A low subtype than in the AR-A high or average subtype (Table 6).

## 4. Discussion

Minimizing toxicity and normalizing testosterone levels while preserving HRQoL and clinical efficacy is a central goal of BCR PC treatment. To reach this goal, intermittent ADT over 6- to 12-month intervals has been the standard of care in this clinical setting and has been shown to be non-inferior with respect to overall survival compared with continuous ADT [12, 13]. The next-generation AR inhibitor apalutamide combined with ongoing ADT has been shown to improve clinical outcomes and maintain HRQoL in patients with advanced disease [14, 15, 29, 30], but its treatment effect with or without ADT in BCR PC is unknown. Additionally, the utility of a finite period (12 months) of apalutamide monotherapy for BCR PC has not been previously evaluated in a randomized fashion. We found that 12 months of treatment with apalutamide plus ADT produced no notable difference in HRQoL at 12 and 24 months from that with ADT monotherapy, although the limited sample size and resultant wide CI precluded confirmation of statistical noninferiority. Compared with ADT alone, apalutamide plus ADT resulted in a higher rate of achieved PSA <0.2 ng/mL at 7 months and similar time to testosterone recovery. This observation is intriguing in the context of previous findings showing that patients achieving PSA ≤0.2 ng/mL after 7-month induction of ADT had better survival than those achieving PSA >0.2 ng/mL [21]. Apalutamide plus ADT also appeared to prolong time to PSA progression and increase the rate of testosterone recovery without PSA progression at 24 months over ADT alone, although a statistically significant difference could not be demonstrated. These hypothesis-generating data provide support for further evaluating treatment with apalutamide plus ADT over ADT monotherapy in BCR PC as the optimal duration of therapy remains to be determined. The ongoing randomized, phase 3 AFT-19 study seeks to validate these findings [16].

The CI for HRQoL at 12 months exceeded the noninferiority margin of ≥7-point mean change difference in total FACT-P score between apalutamide plus ADT and apalutamide monotherapy. We chose a noninferiority margin of −7, which is within the range of clinically meaningful change of 6 to 10 points reported for advanced PC [23] and 5 to 9 points for other cancer types [24, 25]. The limited sample size of 30 patients per treatment group led to wide CIs in the noninferiority analysis, likely contributing to the failure to meet the prespecified noninferiority cut-off point. Subsequent studies with larger sample sizes and greater statistical power will be required to definitively compare ADT with or without apalutamide with respect to the quality-of-life outcomes.

Intermittent or finite ADT treatment of advanced or localized PC has been assessed in the past [31–33], but the optimal duration of treatment has not been established. Intermittent ADT has been shown to be non-inferior to continuous therapy in terms of survival in advanced cancer after a 3-month induction and is associated with better sexual activity [31]. In patients with indolent localized disease, 3-month ADT has been shown to result in nearly 50% of patients continuing to have negative biopsies [32]. With regard to HRQoL, 4-month ADT resulted in significantly higher FACT-P total scores compared with the 10-month treatment [33]. We demonstrated that apalutamide given with ADT for 12 months prolonged time to PSA progression off therapy without any increase in testosterone recovery time or impact on HRQoL. Whether apalutamide plus ADT with a shorter treatment duration would maintain these results needs to be assessed in the future.

Non-castrating apalutamide monotherapy did not improve HRQoL at 12 and 24 months and was associated with a shorter time to PSA progression compared with ADT alone. The difference in change from baseline in FACT-P score was not clinically meaningful between the apalutamide monotherapy and ADT monotherapy arms. The longer time to PSA progression with ADT may reflect the time required for testosterone recovery. While there was a subset of 10 patients in the apalutamide monotherapy group without PSA progression at 12 months after cessation of treatment, current predictive biomarkers are unable to identify which subset of patients might achieve a similar response. An overall lack of favorable improvement in HRQoL and shorter median time to PSA progression, along with the lack of molecular identifiers, temper any enthusiasm of non-castrating peripheral androgen blockade in this patient population.

Safety findings of apalutamide plus ADT were consistent with the known safety profile of apalutamide combination treatment [14, 15, 34]. Gynecomastia and nipple pain, common with antiandrogen monotherapy use, were more frequent with apalutamide monotherapy and could be mitigated by prophylactic breast radiation and/or tamoxifen [35], allowed per protocol, although only one patient received irradiation and none received tamoxifen. Gynecomastia and nipple pain were observed less frequently with the addition of ADT to apalutamide. The lower incidence of falls with apalutamide monotherapy (3.4%) than with apalutamide plus ADT (12.9%) is notable, but the analysis is hampered by the small sample size.

Ephrin receptor *EPHA3* expression has been associated with tumorigenicity in vitro [36] and has been shown to be an independent prognostic indicator of poor survival [37]. The apparent role of *EPHA3* in our study as a negative predictor of hormonal therapy response requires further investigation. AR-A low and GC high subtypes have been associated with a high risk of recurrent disease [38, 39], and the basal subtypes have been associated with aggressive disease and low sensitivity to ADT [40, 41]. The small sample size precluded any meaningful analysis; therefore, the role of molecular subtypes in predicting outcomes in BCR PC needs further confirmation in a larger sample.

Limitations of this study include the open-label design and relatively small sample size, precluding definitive comparisons of FACT-P or efficacy endpoints between groups. Larger studies with longer follow-up will be needed to definitively test whether the addition of apalutamide to ADT improves outcomes without a detrimental effect on quality of life. Another significant limitation of the current study is the under-representation of minority patient populations, including Black patients. Inequities in clinical study access are being addressed in follow-on studies in the BCR patient population via inclusion of clinical sites with more diverse catchment areas. Enrolled patients were metastasis-free based on conventional imaging. More sensitive next-generation imaging (e.g., using prostate-specific membrane antigen) was not available when this study was designed; therefore, the optimal systemic treatment regimen coupled with current imaging techniques remains to be elucidated. The results of randomized phase 2 studies supporting metastasis-directed therapy in oligometastatic disease identified on positron emission tomography [42, 43] are eagerly awaited.

## 5. Conclusions

In this hypothesis-generating BCR PC study, non-castrating apalutamide monotherapy was not superior to ADT whereas apalutamide plus ADT did not demonstrate a statistically significant noninferiority in change from baseline in overall HRQoL. As expected, apalutamide monotherapy did not cause testosterone suppression and, as a result, time to PSA progression after therapy cessation was shorter than that with ADT or apalutamide plus ADT treatment. The aggregated efficacy and safety outcomes support further evaluation of apalutamide plus ADT in BCR PC. Further evaluation of *EPHA3* as a potential biomarker of AR signaling inhibition in BCR PC is warranted.

## Figures and Tables

**Table 1:** Inclusion and exclusion criteria in the ARN-509-002 study.

Inclusion criteria	
1	Patients aged ≥18 years with BCR PC and PSADT ≤12 months after radical prostatectomy and/or radiation therapy undertaken with curative intent
2	No evidence of metastatic disease on conventional imaging consisting of computed tomography or magnetic resonance imaging of the abdomen/pelvis and whole-body nuclear bone scan
3	Prior primary or salvage radiation or not a candidate for localized salvage radiation based on patient preference or physician discretion
4	Minimum PSA of 1.0 ng/mL in patients who received prior radical prostatectomy with or without adjuvant or salvage radiation or PSA nadir plus 2.0 ng/mL in patients who had definitive radiation therapy without prior radical prostatectomy
5	Serum testosterone level ≥150 ng/dL
6	Eastern cooperative oncology group performance status of 0 or 1

Exclusion criteria	
1	Treatment with an oral antiandrogen within 6 weeks prior to randomization
2	Prior treatment with ADT for BCR PC. ADT with or without prior local definitive and/or salvage therapy was allowed provided the last dose of ADT was >6 months before study entry and the screening serum testosterone was ≥150 ng/dL
3	Treatment with 5-alpha reductase antagonist within 6 weeks prior to randomization
4	Prior bilateral orchiectomy

**Table 2:** Prespecified endpoints in the ARN-509-002 study.

Primary endpoint	
1	Mean change from baseline to 12 months in FACT-P total score^*∗*^

Secondary endpoints	
1	Mean change from baseline in EORTC QLQ-C30/QLQ-PR25 over time
2	Mean change from baseline in SHIM over time
3	Time to PSA progression^†^

Exploratory endpoints	
1	Proportion of patients without evidence of PSA or radiographic progression in the setting of recovered serum testosterone (≥150 ng/dL) at 24 months
2	Proportion of patients with PSA <0.2 ng/mL after 7 months of therapy^‡^
3	Time to testosterone recovery >150 ng/dL during the off-therapy observation period
4	Mean change from baseline in bone mineral density at 12 months^§^

^*∗*^Treatment period of 12 months was selected based on previously published studies of intermittent ADT that used, in general, an induction period of 8 to 12 months [12,13]. ^†^Time to PSA progression was defined as PSA rise to ≥50% of the baseline serum PSA or rise of ≥2 ng/mL above the nadir, whichever was higher, confirmed by repeat measurement at least 2 weeks later. ^‡^Based on a prior clinical study of men with mCSPC that showed that after 7 months of ADT induction, men with a nadir PSA level of <0.2 ng/mL, versus 0.2–4.0 ng/mL, or versus >4.0 ng/mL had progressively shorter median overall survival [21]. ^§^The endpoint of mean change from baseline in bone mineral density at 12 months was based on a clinical study that found a significant decrease in bone mineral density after 12 months of ADT [22].

**Table 3:** Demographic and baseline characteristics (intent-to-treat population).

Baseline patient characteristic	Apalutamide + ADT (*n* = 31)	Apalutamide (*n* = 29)	ADT (*n* = 30)
Median age, years (range)	67.0 (54–78)	66.0 (55–79)	68.5 (46–80)
Race			
White	29 (93.5%)	26 (89.7%)	26 (86.7%)
Asian	1 (3.2%)	1 (3.4%)	2 (6.7%)
Black or African American	1 (3.2%)	1 (3.4%)	0
Unknown	0	1 (3.4%)	2 (6.7%)
Median time from initial diagnosis to randomization, years (range)	6.1 (0.9–22.0)	5.7 (0.6–15.7)	6.0 (2.2–15.3)
ECOG performance status			
0	30 (96.8%)	27 (93.1%)	24 (80.0%)
1	1 (3.2%)	2 (6.9%)	6 (20.0%)
Tumor stage at initial diagnosis			
T1	0	1 (3.4%)	0
T1C	5 (16.1%)	2 (6.9%)	3 (10.0%)
T2	1 (3.2%)	1 (3.4%)	2 (6.7%)
T2A	1 (3.2%)	4 (13.8%)	1 (3.3%)
T2B	2 (6.5%)	3 (10.3%)	0
T2C	9 (29.0%)	8 (27.6%)	11 (36.7%)
T3	1 (3.2%)	1 (3.4%)	0
T3A	5 (16.1%)	3 (10.3%)	7 (23.3%)
T3B	7 (22.6%)	6 (20.7%)	6 (20.0%)
Gleason score at initial diagnosis			
*n*	31	27	29
≤7	20 (64.5%)	16 (59.3%)	20 (66.7%)
≥8	11 (35.5%)	11 (40.7%)	9 (31.0%)
Median PSA at randomization, *µ*g/L (range)	4.1 (1.2–38.8)	2.7 (1.0–42.3)	4.0 (1.2–29.8)
PSA doubling time			
<6 months	21 (67.7%)	20 (69.0%)	19 (63.3%)
≥6 months	10 (32.3%)	9 (31.0%)	11 (36.7%)
Risk categories^∗^			
Low	0	3 (10.3%)	1 (3.3%)
Intermediate	15 (48.4%)	14 (48.3)	15 (50.0%)
High	16 (51.6%)	12 (41.4%)	14 (46.7%)
Prior radical prostatectomy^†^	28 (90.3%)	23 (79.3%)	24 (80.0%)
Prior radiation therapy^†^	25 (80.6%)	25 (86.2%)	29 (96.7%)
Primary	4 (12.9%)	6 (20.7%)	5 (16.7%)
Salvage	20 (64.5%)	18 (62.1%)	22 (73.3%)
Other	3 (9.7%)	2 (6.9%)	2 (6.7%)
Adjuvant	2 (6.5%)	2 (6.9%)	1 (3.3%)
Pretreatment for brachytherapy	1 (3.2%)	0	0
Metastatic disease/palliative	0	0	1 (3.3%)
Radical prostatectomy and salvage radiation therapy	18 (58.1%)	17 (58.6%)	21 (70.0%)
Baseline FACT-P total score^‡^			
*n*	30	28	28
Median	131.2	125.0	127.0
Range	82.0–144.0	81.8–150.0	92.7–150.0

^∗^ Based on European Association of Urology Prostate Cancer guidelines [26]. ^†^ Patients with multiple therapies were counted only once. ^‡^The scoring range of the FACT-P total score for each patient is 0–156, with higher scores indicating better HRQoL and higher treatment tolerability. ECOG :  Eastern Cooperative Oncology Group.

**Table 4:** Summary of serum testosterone recovery (*T* > 150 ng/dL) at 24 months with or without PSA progression.

	Apalutamide + ADT (*n* = 29)	Apalutamide (*n* = 27)	ADT (*n* = 26)
Patients who achieved testosterone >150 ng/dL^*∗*^	17 (58.6%)	24 (88.9%)	14 (53.8%)
Median time to serum testosterone recovery, months	24.0	12.1	23.3
(Range)	(10.9–32.8)	(11.4–24.0)	(6.4–25.2)
Serum testosterone >150 ng/dL with PSA progression	6 (20.7%)	14 (51.9%)	9 (34.6%)
Serum testosterone >150 ng/dL without PSA progression^†^	11 (37.9%)	10 (37.0%)	5 (19.2%)

^*∗*^Includes a serum testosterone value > 150 ng/dl from start of treatment up to and including 24 months. ^†^Proportion of patients in each treatment group without PSA or radiographic progression.

**Table 5:** Summary of treatment-emergent adverse events in the safety population.

	Apalutamide + ADT (*n* = 31)	Apalutamide (*n* = 29)	ADT (*n* = 29)
Any treatment-related AE	31 (100%)	29 (100%)	28 (96.6%)
Serious AE	5 (16.1%)	0	3 (10.3%)
AE leading to death	0	^0^	1 (3.4%)^*∗*^
AE leading to discontinuation of study agent or termination of study participation	0	2 (6.9%)	1 (3.4%)
Grade ≥3 AEs	9 (29.0%)	5 (17.2%)	4 (13.8%)
Drug-related grade ≥3 AEs	2 (6.5%)	2 (6.9%)	0
Most frequently reported AEs occurring in ≥25% of patients			
Fatigue	24 (77.4%)	19 (65.5%)	22 (75.9%)
Hot flush	26 (83.9%)	9 (31.0%)	25 (86.2%)
Arthralgia	7 (22.6%)	8 (27.6%)	5 (17.2%)
Gynecomastia	1 (3.2%)	12 (41.4%)	3 (10.3%)
Nipple pain	0	12 (41.4%)	0
Insomnia	10 (32.3%)	1 (3.4%)	2 (6.9%)
AEs of special interest	11 (35.5%)	11 (37.9%)	5 (17.2%)
Rash^†^	6 (19.4%)	10 (34.5%)	3 (10.3%)
Fall	4 (12.9%)	1 (3.4%)	2 (6.9%)
Hypothyroidism	2 (6.5%)	1 (3.4%)	0
Fracture^‡^	1 (3.2%)	0	1 (3.4%)

^*∗*^Patient experienced a fatal event of toxic epidermal necrolysis within 30 days of the last dose of ADT; it was not considered related to the study treatment. ^†^Grouped term; includes rash, rash pruritic, rash maculo-papular, conjunctivitis, rash generalized, rash papular, stomatitis, and toxic epidermal necrolysis. ^‡^Grouped term; includes fracture pain, hand fracture, rib fracture.

**Table 6:** PSA progression in patients with various molecular subtypes.

	AR-A		PAM50			GC score	Total
	Low	High or average	Basal	Luminal	Low to average	High	
N	9 (34.6%)	17 (65.4%)	15 (57.7%)	11 (42.3%)	11 (42.3%)	15 (57.7%)	26 (100%)
Time to PSA progression, months							
Mean (SD)	36.4 (9.4)	29.0 (7.0)	31.8 (9.9)	31.2 (6.6)	34.1 (9.9)	29.7 (7.1)	31.6 (8.5)
Median	36.6	30.2	32.9	32.8	33.6	32.9	32.9
Range	20.3–56.6	17.7–39.4	17.8–56.6	17.7–39.4	17.8–56.6	17.7–38.6	17.7–56.6

PAM50: prediction analysis of microarray 50; SD: standard deviation.

## Data Availability

The data sharing policy of Janssen Pharmaceutical Companies of Johnson & Johnson is available at https://www.janssen.com/clinical-trials/transparency. As noted on this site, requests for access to the study data can be submitted through Yale Open Data Access (YODA) Project site at https://yoda.yale.edu. The sponsor commissioned an independent data and safety monitoring committee to review safety data and the results of the primary efficacy analysis. Data were transcribed by study personnel at each clinical site from source documents into electronic case report forms prepared by the sponsor.

## Supplementary Materials

Supplementary Materials The supplementary material includes additional methods and results: *S1*. Supplementary methods; *S2*. Supplementary results and supplemental references; *Table S1*. Differences in estimated LS mean change from baseline (95% CI) in EORTC QLQ-C30/QLQ-PR25 and SHIM between treatment groups; *Table S2.* Median change from baseline in bone mineral density at 12 months; *Table S3.* Expression of biomarkers from whole-blood RNA and their association with PSA progression. ARv7 and biomarkers detected in ≥10% of ARN-509-002 patients at baseline are shown; *Figure S1.* FACT-P total score over time.
