# Supplementary material for: Randomized, Open-Label Phase 2 Study of Apalutamide plus Androgen Deprivation Therapy versus Apalutamide Monotherapy versus Androgen Deprivation Monotherapy in Patients with Biochemically Recurrent Prostate Cancer
Source: Prostate Cancer. 2022 Sep 28;2022:5454727. doi: 10.1155/2022/5454727 (PMC9534720; doi:10.1155/2022/5454727)

**Supplementary Material for** **Randomized, Open-Label Phase 2 Study of Apalutamide Plus Androgen Deprivation Therapy Versus Apalutamide Monotherapy Versus Androgen Deprivation Monotherapy in Patients with Biochemically Recurrent Prostate Cancer**

Rahul Aggarwal, Joshi J. Alumkal, Russell Z. Szmulewitz, Celestia S. Higano, Alan H. Bryce, Angela Lopez-Gitlitz, Sharon A. McCarthy, Branko Miladinovic, Kelly McQuarrie, Shibu Thomas, Ke Zhang, and Eric J. Small

Table of Contents

[S1. Supplementary Methods 2](#_Toc113896836)

[S2. Supplementary Results 6](#_Toc113896837)

[Supplementary References 7](#_Toc113896838)

[Table S1. Differences in estimated LS mean change from baseline (95% CI) in EORTC QLQ-C30/QLQ-PR25 and SHIM between treatment groups. 9](#_Toc113896839)

[Table S2. Median change from baseline in bone mineral density at 12 months. 12](#_Toc113896840)

[Table S3. Expression of biomarkers from whole-blood RNA and their association with PSA progression. ARv7 and biomarkers detected in ≥10% of ARN-509-002 patients at baseline are shown. 13](#_Toc113896841)

[Figure S1. FACT-P total score over time. 14](#_Toc113896842)

# S1. Supplementary Methods

S1.1. *Study Design and Patients.* Balance in treatment assignment was achieved using a randomized block design. Randomization was carried out via computer-generated random assignment. All patients commenced treatment within 5 calendar days of randomization.

The study was conducted in accordance with current International Conference on Harmonisation guidelines for Good Clinical Practice and principles set forth in the Declaration of Helsinki.

*S1.2. Outcomes.* The Functional Assessment of Cancer Therapy-Prostate (FACT-P) is a 39-item questionnaire consisting of a 27-item core health-related quality of life (HRQoL) measure grouped into physical well-being, social/family well-being, emotional well-being, and functional well-being subscales and 12 additional items specific to prostate cancer (PC). The total score ranges from 0 to 156, and higher scores indicate a higher degree of functioning and better HRQoL. The FACT-P is an established HRQoL instrument used in clinical studies of patients with advanced PC [1].

The European Organization for Research and Treatment of Cancer (EORTC) Quality of Life Questionnaire-C30 (QLQ-C30) is a validated [2], widely used scale to measure HRQoL in patients with PC that consists of 30 general items grouped into five multi-item functional scales (physical function, role function, emotional function, social function, and global health status/HRQoL) and nine single-item scales, including fatigue, pain, dyspnea, and gastrointestinal problems. The EORTC QLQ Prostate Cancer Module (PR25) is a well-validated scale that includes 25 PC-specific items [3]. All scales are linearly transformed from 0 to 100.

The Sexual Health Inventory for Men (SHIM), a well-validated abridged five-item version of the 15-item International Index of Erectile Function, consists of five items pertaining to sexual functioning, with scores for most items ranging from 0 to 5 [4].

*S1.3. Procedures.* Choice of ADT and dosing schedule was at the discretion of the individual investigators. Serum PSA was measured at baseline and monthly for up to 12 months during the on-treatment period and every 2 months during the off-treatment period up to 24 months or progression, defined as PSA or radiographic progression. Serum testosterone levels were measured at 3, 6, and 12 months during the on-treatment period and every 2 months during the off-treatment period up to 24 months or progression. FACT-P, EORTC QLQ-C30/QLQ-PR25, and SHIM questionnaires were assigned at baseline, 3, 12, and 24 months or progression. A whole-body nuclear bone scan and computed tomography/magnetic resonance imaging of the abdomen/pelvis were obtained as prompted by signs or symptoms of metastatic disease and at PSA progression. Dual energy x-ray absorptiometry scan measuring bone mineral density in the femoral neck and lumbar spine was assessed at baseline and 12 months or progression. Radiographic progression, defined as detection of new metastases on either bone scan or cross-sectional imaging (computed tomography or magnetic resonance imaging), was assessed by investigators (no central review).

*S1.4. Biomarker Analysis.* Two exploratory biomarker analyses were conducted in a subset of patients. First, archival tumor samples or slides collected at baseline were categorized based on three molecular classifiers: 1) androgen receptor activity low vs. moderate to high (≤11 versus >11, respectively) [5]; 2) DECIPHER^®^ genomic classifier high versus low to average (>0.6 versus ≤0.6) [6]; and 3) PAM50 basal versus luminal subtypes [7,8]. Gene expression profiling of archival samples was performed using DECIPHER^®^ human exon assay (GenomeDX, Inc., San Diego, CA). Second, whole-blood samples at baseline and end-of-study treatment (EOST; 12 months of treatment or progression) were assessed using a custom biomarker panel consisting of 36 markers that were selected from >200 poor prognosis, prostate cancer biomarkers based on differential expression and specificity/sensitivity cut-offs. Total whole-blood RNA was extracted from whole blood and transcribed to complementary DNA using random hexamers. Quantitative real-time polymerase chain reaction was used to detect biomarkers in four replicates using the Biomark Fluidigm Dynamic Array microfluidic system (Expression Analysis, Durham, NC). Means of replicates were summarized as positive (≤35-cycle threshold) or negative (>35-cycle threshold) detection.

*S1.5. Statistical Analysis.* Approximately 30 patients were planned for randomization to each treatment group. This sample size had 80% power to detect a difference of 7.5 points in the mean change from baseline in FACT-P total score at 12 months between apalutamide monotherapy and ADT monotherapy (superiority assessment), assuming a common standard deviation of the change in each group of 8.7. A change in FACT score that is clinically meaningful and impacts treatment in prior studies ranges from 6 to 10 for advanced prostate cancer [9] and 5 to 9 points for other cancer types [10, 11]. The planned sample size also had 82% power for a noninferiority assessment of the mean change in FACT-P total score from baseline at 12 months between apalutamide plus ADT and ADT monotherapy, assuming a common standard deviation of the change in each group of 8.7 and effect size of 0.81. Inferior HRQoL was predefined as a ≥7-point difference in mean change in FACT-P score between treatment groups. Both assessments were based on a two-group *t* test, using a two-sided level of significance of 0.025 (adjusted for two comparisons). To provide unbiased estimates, linear mixed models were used for analysis of the primary endpoint assuming that missing data occur at random.

The formal control of type 1 error with multiplicity adjustment was not planned for the secondary endpoints.

The mean change from baseline in total FACT-P, EORTC QLQ-C30/QLQ-PR25, and SHIM scores at 3, 12 and 24 months were analyzed using a mixed model for repeated measures. Change from baseline was estimated using least squares (LS) means, with factor levels weighted according to overall baseline sample means and stratification factors of PSA doubling time (<6 versus 6–12 months) and age (≤70 versus >70 years). Time to PSA progression was assessed using the Kaplan-Meier method, stratified proportional hazards model, and log-rank test. Testosterone recovery was assessed using the Kaplan-Meier method and stratified Cochran-Mantel-Haenszel test. Quality of life and PSA suppression endpoints were determined in the intent-to-treat population, defined as all randomized patients. Testosterone recovery was summarized at 1, 6, and 12 months of therapy and 18 and 24 months off therapy in the modified intent-to-treat population, defined as all randomized patients except those who withdrew from the study prior to 24 months. In the off-therapy observation period, testosterone levels from the highest recorded testosterone measurement closest to 18 and 24 months were included. Bone mineral density in the femoral neck and lumbar spine was assessed in the intent-to-treat population and was compared using an analysis of covariance that controls for baseline values of corresponding measurement. All *P* values except for the primary superiority test were nominal.

Correlation between time to PSA progression and biomarker status at baseline was assessed using Cox proportional hazards model. All statistical analyses were performed according to a prespecified statistical analysis plan using common statistical methods and SAS 9.4.

Treatment-emergent adverse events (TEAEs) were assessed in the safety population, defined as patients who received at least one dose of study drug, and were graded according to the National Cancer Institute Common Terminology Criteria for Adverse Events version 4.03.

# S2. Supplementary Results

*S2.1.* *EORTC QLQ-C30/QLQ-PR25 and SHIM.* For constipation, financial difficulties, and sexual activity, differences in LS mean change from baseline (95% CI) at 12 months versus ADT were −2.4 (−10.9 to 6.0), 10.2 (1.0–19.4), and −8.5 (−19.3 to 2.3) for the apalutamide plus ADT group and −9.9 (−18.8 to −0.9), 10.2 (0.4–20.1), and −14.3 (−25.6 to −3.0) for the apalutamide group, respectively. Differences in estimated LS mean change from baseline (95% CI) in SHIM at 12 months versus ADT were 0.6 (−1.64 to 2.9) for the apalutamide group and −0.3 (−2.5 to 2.0) for the apalutamide plus ADT group.

S2.2. *Dose reductions and Interruptions.* Dose reductions due to TEAEs occurred in one patient in the apalutamide plus ADT group and in three patients in the apalutamide group. The most frequent TEAEs leading to dose reduction reported for >1% of patients were skin and subcutaneous tissue disorders (6.9% of patients in the apalutamide group and 0% of patients in each of the other groups) and a group term of general disorders and administration site conditions (3.2% of patients in the apalutamide plus ADT group and 0% of patients in each of the other groups). Dose interruptions occurred in 6 patients in the apalutamide plus ADT group (one to two interruptions; 19.4%) and in three patients in the apalutamide group (three to five interruptions; 10.3%). In the apalutamide plus ADT group, five (16.1%) patients had one dose interruption and one (3.2%) had two dose interruptions. In the apalutamide group, three patients had dose interruptions: one had three interruptions, one had four interruptions, and one had five interruptions.

# Supplementary References

1. P. Esper, F. Mo, G, Chodak, et al. “Measuring quality of life in men with prostate cancer using the Functional Assessment of Cancer Therapy-Prostate instrument,” *Urology,* vol. 50, no. 6, pp. 920-928, 1997.
2. G. van Andel, A. Bottomley, S. D. Fossa, et al. “An international field study of the EORTC QLQ-PR25: a questionnaire for assessing the health-related quality of life of patients with prostate cancer,” *European Journal of Cancer,* vol. 44, no. 16, pp. 2418–2424, 2008.
3. R. C, Rosen, J. C. Cappelleri, M. D. Smith et al. “Development and evaluation of an abridged, 5-item version of the International Index of Erectile Function (IIEF-5) as a diagnostic tool for erectile dysfunction,” *International Journal of Impotence Research*, vol. 11, no. 6, pp. 319–326, 1999.
4. M. Hussain, C. M. Tangen, C. Higano, et al. “Absolute prostate-specific antigen value after androgen deprivation is a strong independent predictor of survival in new metastatic prostate cancer: data from Southwest Oncology Group Trial 9346 (INT-0162),” *Journal of Clinical Oncology*, vol. 24, no. 24, pp. 3984-3990, 2006.
5. D. E, Spratt, M. Alshalalfa, N. Fishbane, et al. “Transcriptomic heterogeneity of androgen receptor activity defines a de novo low AR-active subclass in treatment naive primary prostate cancer,” *Clinical Cancer Research,* vol. 25, no. 22, pp. 6721–6730, 2019.
6. S. G. Zhao, S. L. Chang, N. Erho, et al. “Associations of luminal and basal subtyping of prostate cancer with prognosis and response to androgen deprivation therapy,” *JAMA Oncol*ogy, vol. 3, no. 3, pp. 1663–1672, 2017.
7. D. Zhang, D. Park, Y. Zhong, et al. “Stem cell and neurogenic gene-expression profiles link prostate basal cells to aggressive prostate cancer,” *Nature Communications,* vol. 7, no. 7, p. 10798, 2016.
8. D. Cella, M. B. Nochol, D. Eton, et al. “Estimating clinically meaningful changes for the Functional Assessment of Cancer Therapy--Prostate: results from a clinical trial of patients with metastatic hormone-refractory prostate cancer,” *Value in Health,* vol. 12, no. 1, pp. 124–129, 2009.
9. J. L. Steel, D. T. Eton, D. Cella, et al. “Clinically meaningful changes in health-related quality of life in patients diagnosed with hepatobiliary carcinoma,” *Annals of Oncology,* vol. 17, no. 2, pp. 304–312, 2006.
10. D. Cella, D. T. Eton, D. L. Fairclough, et al, “What is a clinically meaningful change on the Functional Assessment of Cancer Therapy-Lung (FACT-L) Questionnaire? Results from Eastern Cooperative Oncology Group (ECOG) Study 5592,” *Journal of Clinical Epidemiology*, vol. 55, no. 3, pp. 285–295, 2002.

# Table S1. Differences in estimated LS mean change from baseline (95% CI) in EORTC QLQ-C30/QLQ-PR25 and SHIM between treatment groups.

| Difference in LS mean | Apalutamide + ADT vs ADT | Apalutamide vs ADT |
| --- | --- | --- |
| EORTC QLQ-C30/QLQ-PR25 | | |
| Appetite loss |  |  |
| 3 months | 5.24 (−2.02 to 12.50) | 0.63 (*−*6.96 to 8.22) |
| 12 months | 2.21 (*−*6.62 to 11.04) | 1.38 (*−*8.05 to 10.81) |
| 24 months | 7.57 (*−*2.77 to 17.91) | *−*0.21 (*−*11.79 to 11.37) |
| Cognitive functioning |  |  |
| 3 months | *−*5.29 (*−*14.73 to 4.15) | *−*4.39 (*−*14.23 to 5.45) |
| 12 months | *−*6.07 (*−*15.01 to 2.88) | *−*9.30 (*−*18.77 to 0.17) |
| 24 months | 0.66 (*−*9.98 to 11.30) | *−*7.64 (*−*19.53 to 4.25) |
| Constipation |  |  |
| 3 months | 0.55 (*−*7.18 to 8.28) | *−*6.11 (*−*14.13 to 1.91) |
| 12 months | *−*2.43 (*−*10.86 to 6.00) | **−9.85 (−18.79 to −0.91)** |
| 24 months | *−*0.55 (*−*10.93 to 9.82) | 0.46 (*−*11.25 to 12.17) |
| Diarrhea |  |  |
| 3 months | 2.09 (*−*8.84 to 13.03) | 2.11 (*−*9.30 to 13.53) |
| 12 months | 3.26 (*−*6.41 to 12.94) | 0.95 (*−*9.29 to 11.19) |
| 24 months | 4.73 (*−*6.62 to 16.08) | *−*1.61 (*−*14.63 to 11.40) |
| Dyspnea |  |  |
| 3 months | 6.43 (*−*4.78 to 17.64) | 6.80 (*−*4.91 to 18.51) |
| 12 months | 5.04 (*−*5.73 to 15.81) | 5.87 (*−*5.57 to 17.30) |
| 24 months | *−*3.82 (*−*17.57 to 9.93) | *−*2.28 (*−*17.73 to 13.17) |
| Emotional functioning |  |  |
| 3 months | 2.22 (*−*4.93 to 9.37) | −1.78 (−9.23 to 5.68) |
| 12 months | 2.46 (*−*4.05 to 8.96) | 0.33 (*−*6.56 to 7.21) |
| 24 months | 3.73 (*−*4.79 to 12.25) | 4.19 (*−*5.48 to 13.86) |
| Fatigue |  |  |
| 3 months | 11.00 (*−*0.08 to 22.08) | 10.45 (*−*1.37 to 22.28) |
| 12 months | 4.51 (*−*5.93 to 14.95) | *−*2.00 (*−*13.27 to 9.27) |
| 24 months | *−*5.52 (*−*17.98 to 6.94) | *−*5.80 (*−*20.34 to 8.75) |
| Financial difficulties |  |  |
| 3 months | 8.39 (*−*0.33 to 17.11) | 4.12 (*−*4.95 to 13.19) |
| 12 months | **10.16 (0.95–19.36)** | **10.24 (0.42–20.06)** |
| 24 months | 6.79 (*−*4.57 to 18.15) | 9.13 (*−*3.72 to 21.98) |
| Global health status |  |  |
| 3 months | *−*5.62 (*−*14.46 to 3.21) | *−*6.73 (*−*16.01 to 2.55) |
| 12 months | *−*1.18 (*−*9.30 to 6.94) | *−*3.39 (*−*12.02 to 5.25) |
| 24 months | 1.09 (*−*9.32 to 11.51) | 4.41 (*−*7.54 to 16.36) |
| Insomnia |  |  |
| 3 months | 7.47 (*−*8.39 to 23.33) | *−*1.57 (*−*18.15 to 15.02) |
| 12 months | *−*7.29 (*−*21.72 to 7.15) | **−13.19 (−28.50 to 2.13)** |
| 24 months | **−19.34 (−36.93 to −1.74)** | *−*13.85 (*−*34.22 to 6.52) |
| Nausea and vomiting |  |  |
| 3 months | 1.11 (*−*2.88 to 5.09) | 0.74 (*−*3.46 to 4.93) |
| 12 months | 1.44 (*−*2.10 to 4.97) | *−*0.03 (*−*3.82 to 3.76) |
| 24 months | 0.21 (*−*3.88 to 4.31) | *−*1.08 (*−*5.74 to 3.58) |
| Pain |  |  |
| 3 months | 0.96 (*−*9.41 to 11.33) | *−*0.94 (*−*11.72 to 9.83) |
| 12 months | 8.17 (*−*2.14 to 18.48) | *−*1.83 (*−*12.74 to 9.07) |
| 24 months | 0.15 (*−*12.06 to 12.36) | *−*0.72 (*−*14.56 to 13.11) |
| Physical functioning |  |  |
| 3 months | *−*2.19 (*−*8.63 to 4.24) | **−6.04 (−12.75 to 0.67)** |
| 12 months | *−*1.88 (*−*8.35 to 4.60) | *−*1.41 (*−*8.26 to 5.43) |
| 24 months | 3.27 (*−*4.25 to 10.79) | 3.46 (*−*4.98 to 11.91) |
| Role functioning |  |  |
| 3 months | *−*1.22 (*−*11.34 to 8.89) | 2.21 (*−*8.32 to 12.75) |
| 12 months | *−*5.57 (*−*16.17 to 5.03) | *−*3.10 (*−*14.35 to 8.14) |
| 24 months | *−*4.09 (*−*16.68 to 8.50) | 3.56 (*−*10.79 to 17.92) |
| Social functioning |  |  |
| 3 months | *−*3.85 (*−*14.20 to 6.51) | 3.77 (*−*7.01 to 14.55) |
| 12 months | 1.94 (*−*7.82 to 11.70) | 6.58 (*−*3.76 to 16.92) |
| 24 months | *−*8.07 (*−*19.76 to 3.61) | *−*1.25 (*−*14.58 to 12.09) |
| Incontinence aid |  |  |
| 3 months | *−*9.66 (*−*43.61 to 24.29) | *−*18.40 (*−*55.44 to 18.64) |
| 12 months | *−*11.52 (*−*38.35 to 15.30) | 0.81 (*−*26.56 to 28.17) |
| 24 months | *−*1.14 (*−*38.68 to 36.40) | 11.99 (*−*27.26 to 51.24) |
| Bowel symptoms |  |  |
| 3 months | **3.94 (0.04–7.83)** | 2.84 (*−*1.28 to 6.96) |
| 12 months | *−*1.36 (*−*5.48 to 2.76) | *−*3.75 (*−*8.28 to 0.77) |
| 24 months | 2.30 (*−*2.59 to 7.20) | 1.75 (*−*3.92 to 7.43) |
| Hormonal treatment*–*related symptoms | |  |
| 3 months | −2.76 (−3.05 to 8.57) | *−*2.48 (*−*8.51 to 3.56) |
| 12 months | 4.71 (*−*1.76 to 11.18) | 3.33 (*−*3.50 to 10.17) |
| 24 months | *−*1.02 (*−*9.03 to 6.98) | *−*5.12 (*−*14.18 to 3.94) |
| Sexual activity |  |  |
| 3 months | *−*0.40 (*−*11.80 to 11.00) | *−*4.08 (*−*15.85 to 7.68) |
| 12 months | *−*8.48 (*−*19.25 to 2.30) | **−14.34 (−25.64 to −3.03)** |
| 24 months | *−*0.61 (*−*13.93 to 12.70) | *−*1.71 (*−*16.75 to 13.32) |
| Sexual functioning |  |  |
| 3 months | **27.54 (*−*0.42 to 55.51)** | 12.38 (*−*10.40 to 35.17) |
| 12 months | *−*8.36 (*−*39.01 to 22.29) | *−*11.84 (*−*38.93 to 15.26) |
| 24 months | 6.73 (*−*26.72 to 40.18) | 18.74 (*−*15.56 to 53.05) |
| Urinary symptoms |  |  |
| 3 months | *−*1.71 (*−*8.39 to 4.98) | ***−*6.32 (*−*13.33 to 0.69)** |
| 12 months | 5.07 (*−*1.37 to 11.50) | *−*3.66 (*−*10.52 to 3.21) |
| 24 months | 3.30 (*−*4.94 to 11.53) | *−*5.34 (*−*14.61 to 3.93) |
| SHIM | | |
| 3 months | 0.10 (*−*1.79 to 1.98) | **1.84 (*−*0.08 to 3.76)** |
| 12 months | *−*0.26 (*−*2.46 to 1.93) | 0.61 (*−*1.64 to 2.85) |
| 24 months | −0.42 (−3.43 to 2.59) | *−*0.06 (−3.25 to 3.13) |

Bold: significance level ≤0.1.

# Table S2. Median change from baseline in bone mineral density at 12 months.

| Bone mineral density (g/cm^2^),  median (range) | Apalutamide + ADT  (*n* = 31) | Apalutamide  (*n* = 29) | ADT  (*n* = 30) |
| --- | --- | --- | --- |
| Femoral neck | 0 (*−*0.1 to 0) | 0.01 (*−*0.2 to 0.8) | *−*0.01 (*−*0.2 to 0.8) |
| L-spine | *−*0.06 (*−*0.3 to 0) | 0.01 (*−*0.1 to 0.2) | *−*0.03 (*−*0.2 to 0.9) |
| Total hip | *−*0.04 (*−*0.1 to 0) | 0.01 (*−*0.2 to 0.1) | *−*0.01 (0 to 0) |

# Table S3. Expression of biomarkers from whole-blood RNA and their association with PSA progression. ARv7 and biomarkers detected in ≥10% of ARN-509-002 patients at baseline are shown.

| Biomarker | Expression, *n*. | | | Median time to PSA progression by biomarker status at baseline, mo | | |
| --- | --- | --- | --- | --- | --- | --- |
|  | Baseline  (*n* = 40) | EOST  (*n* = 54) | *P* value* | Biomarker positive | Biomarker negative | *P* value |
| ARv7 | 1 (2.5) | 2 (3.7) | 1 | NR | 37.0 | 0.998 |
| *ADAMS1* | 7 (17.5) | 11 (20.4) | 0.796 | 30.4 | 37.0 | 0.461 |
| *C9orf152* | 19 (47.5) | 28 (51.9) | 0.835 | 36.1 | 37.0 | 0.784 |
| *CCBE1* | 4 (10.0) | 5 (9.3) | 1 | NR | 37.0 | 0.872 |
| *CYP3A5* | 17 (42.5) | 25 (46.3) | 0.834 | 36.1 | 37.0 | 0.313 |
| *EPHA3* | 12 (30) | 19 (35.2) | 0.661 | NR | 36.1 | 0.02 |
| *FAM13C* | 4 (10) | 4 (7.4) | 0.719 | 30.4 | 37.0 | 0.947 |
| *FOXA1* | 14 (35) | 18 (33.3) | 1 | NR | 37.0 | 0.951 |
| *KLK4* | 10 (25.0) | 17 (31.5) | 0.645 | NR | 36.1 | 0.595 |
| *LGR5* | 5 (12.5) | 8 (14.8) | 1 | NR | 36.1 | 0.1 |
| *MYBPC1* | 5 (12.5) | 12 (22.2) | 0.284 | NR | 36.1 | 0.37 |
| *NPY* | 7 (17.5) | 14 (25.9) | 0.454 | NR | 36.1 | 0.26 |
| *PGR* | 1 (2.5) | 6 (11.1) | 0.232 | NR | 37.0 | NE |
| *RELN* | 28 (70) | 42 (77.8) | 0.475 | 36.1 | 37.0 | 0.927 |

NE: not estimable; NR: not reached. *Fisher’s exact test for comparison between baseline and EOST.

# Figure S1. FACT-P total score over time.


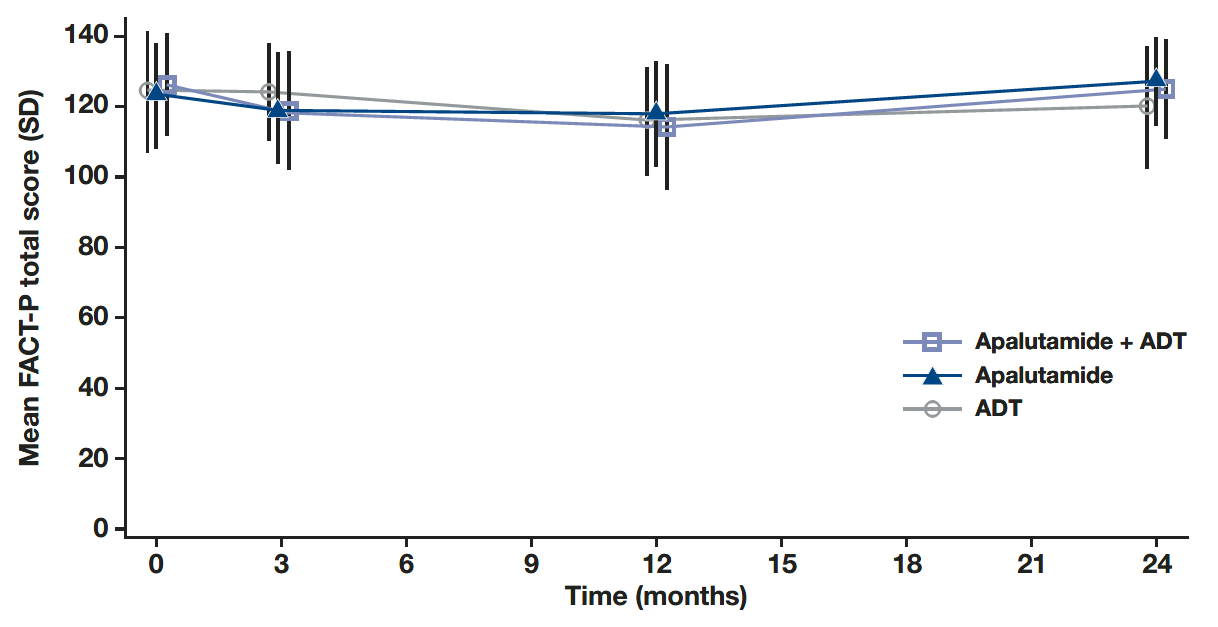

Supplement: Supplementary Materials — The supplementary material includes additional methods and results: S1. Supplementary methods; S2. Supplementary results and supplemental references; Table S1. Differences in estimated LS mean change from baseline (95% CI) in EORTC QLQ-C30/QLQ-PR25 and SHIM between treatment groups; Table S2. Median change from baseline in bone mineral density at 12 months; Table S3. Expression of biomarkers from whole-blood RNA and their association with PSA progression. ARv7 and biomarkers detected in ≥10% of ARN-509-002 patients at baseline are shown; Figure S1. FACT-P total score over time. [file 5454727.f1.docx]
